# Epigenetic inactivation of the *MIR129-2* in hematological malignancies

**DOI:** 10.1186/1756-8722-6-16

**Published:** 2013-02-14

**Authors:** Kwan-Yeung Wong, Rita Lok-Hay Yim, Yok-Lam Kwong, Chung-Ying Leung, Pak-Kwan Hui, Florence Cheung, Raymond Liang, Dong-Yan Jin, Chor-Sang Chim

**Affiliations:** 1 Department of Medicine, Queen Mary Hospital, The University of Hong Kong, Hong Kong, Hong Kong; 2 Department of Pathology, United Christian Hospital, Kowloon, Hong Kong; 3 Department of Pathology, Kwong Wah Hospital, Kowloon, Hong Kong; 4 Department of Pathology, Pamela Youde Nethersole Eastern Hospital, Chai Wan, Hong Kong; 5 Department of Biochemistry, The University of Hong Kong, Hong Kong, Hong Kong

**Keywords:** microRNA, Tumor suppressor, Hypermethylation, *MIR129*, Hematological cancers

## Abstract

**Background:**

*MIR129-2* has been shown to be a tumor suppressor microRNA hypermethylated in epithelial cancers.

**Patients and methods:**

Epigenetic inactivation of *MIR129-2* was studied by methylation-specific PCR (MSP) in 13 cell lines (eight myeloma and five lymphoma), 15 normal controls and 344 primary samples including acute myeloid leukemia (AML), acute lymphoblastic leukemia (ALL), chronic myeloid leukemia (CML), chronic lymphocytic leukemia (CLL), non-Hodgkin’s lymphoma (NHL), multiple myeloma (MM) at diagnosis, MM at relapse/progression, and monoclonal gammopathy of undetermined significance (MGUS). Expression of *MIR129* and its target, *SOX4*, in cell lines was measured before and after hypomethylating treatment and *MIR129* overexpression. *MIR129* expression was correlated with *MIR129-2* methylation status in primary lymphoma samples. Tumor suppressor function of *MIR129* was demonstrated by MTT and trypan blue exclusion assay after *MIR129* overexpression.

**Results:**

The sensitivity of the methylated-MSP was one in 10^3^. Different MSP statuses, including complete methylation, partial methylation, and complete unmethylation, were verified by quantitative bisulfite pyrosequencing. All five lymphoma and seven of eight myeloma cell lines showed complete and partial *MIR129-2* methylation. In primary samples, *MIR129-2* methylation was absent in AML and CML, but detected in 5% ALL, 45.9% CLL, 49.5% MM at diagnosis, and 59.1% NHL. In CLL, *MIR129-2* methylation adversely impacted on survival (p=0.004). In MM, *MIR129-2* methylation increased from 27.5% MGUS to 49.5% MM at diagnosis and 41.5% at relapse/progression (p=0.023). In NHL, *MIR129-2* methylation was associated with *MIR124-1* and *MIR203* methylation (p<0.001), and lower *MIR129* expression (p=0.009). Hypomethylation treatment of JEKO-1, homozygously methylated for *MIR129-2*, led to *MIR129-2* demethylation and *MIR129* re-expression, with downregulation of *SOX4* mRNA. Moreover, *MIR129* overexpression in both mantle cell lines, JEKO-1 and GRANTA-519, inhibited cellular proliferation and enhanced cell death, with concomitant *SOX4* mRNA downregulation.

**Conclusions:**

*MIR129-2* is a tumor suppressive microRNA frequently methylated in lymphoid but not myeloid malignancies, leading to reversible *MIR129-2* silencing. In CLL, *MIR129-2* methylation was associated with an inferior survival. In MM, *MIR129-2* methylation might be acquired during progression from MGUS to symptomatic MM. In NHL, *MIR129-2* methylation might collaborate with *MIR124-1* and *MIR203* methylation in lymphomagenesis.

## Background

DNA methylation, which adds a methyl group to the number 5 carbon of a cytosine ring of a CpG dinucleotide, is catalyzed by DNA methyltransferase [1,2]. Cancers are characterized by a global DNA hypomethylation and locus-specific hypermethyla-tion of tumor suppressor gene (TSG). Based on a pathway-specific approach, multiple TSGs in pathways including cell cycle regulation, Janus kinase/signal transducer and activator of transcription (JAK/STAT) signaling, wingless-type MMTV intergration site family (WNT) signaling, and death-associated protein (DAP) kinase-associated intrinsic tumor suppression, have been shown to be inactivated by gene hypermethylation in leukemia, lymphoma and multiple myeloma (MM) [1,3].

MicroRNAs are short sequences (22–25 nucleotides) of non-coding RNA molecules that regulate a range of biological processes by inducing RNA degradation and/or translation inhibition of targeted mRNAs [4]. Precise microRNA expression is commonly dysregulated in human diseases, including cancers. In carcinogenesis, of these aberrantly expressed microRNAs in malignant cells, those upregulated microRNAs which lead to targeting of tumor suppressor genes are known as oncomiRs. On the other hand, those downregulated microRNAs which originally may inactivate oncogenes are known as tumor suppressive microRNAs [5,6]. Recently, DNA methylation has emerged as an important mechanism in the regulation of microRNA expression, in particular, hypermethylation of microRNA gene promoters may lead to inactivation of tumor suppressive microRNAs in cancers [7].

In human, *MIR129* is transcribed from *MIR129-1* and *MIR129-2* located on chromosome 7q32 and 11p11 respectively. A CpG island is present in the proximity of *MIR129-2* but not *MIR129-1* promoter. Moreover, loss of *MIR129* expression by *MIR129-2* methylation has been reported in gastric, endometrial, and colorectal cancers [8-10], leading to upregulation of oncogenes including cyclin-dependent kinase 6 (*CDK6*) and sex determining region Y-box 4 (*SOX4*) mRNAs, thereby illustrating the tumor suppressive effect of *MIR129*[9-12].

We therefore investigated the role of *MIR129-2* methylation and *MIR129*-mediated tumor suppression in a range of hematological malignancies including acute myeloid leukemia (AML), chronic myelogenous leukemia (CML), acute lymphoblastic leukemia (ALL), chronic lymphocytic leukemia (CLL), non-Hodgkin's lymphoma (NHL), and multiple myeloma (MM), together with monoclonal gammopathy of undetermined significance (MGUS), the precursor stage of MM, and MM at relapse/progression.

## Results

### Methylation-specific PCR: *MIR129-2* methylation in controls and cell lines

**Figure 1:**
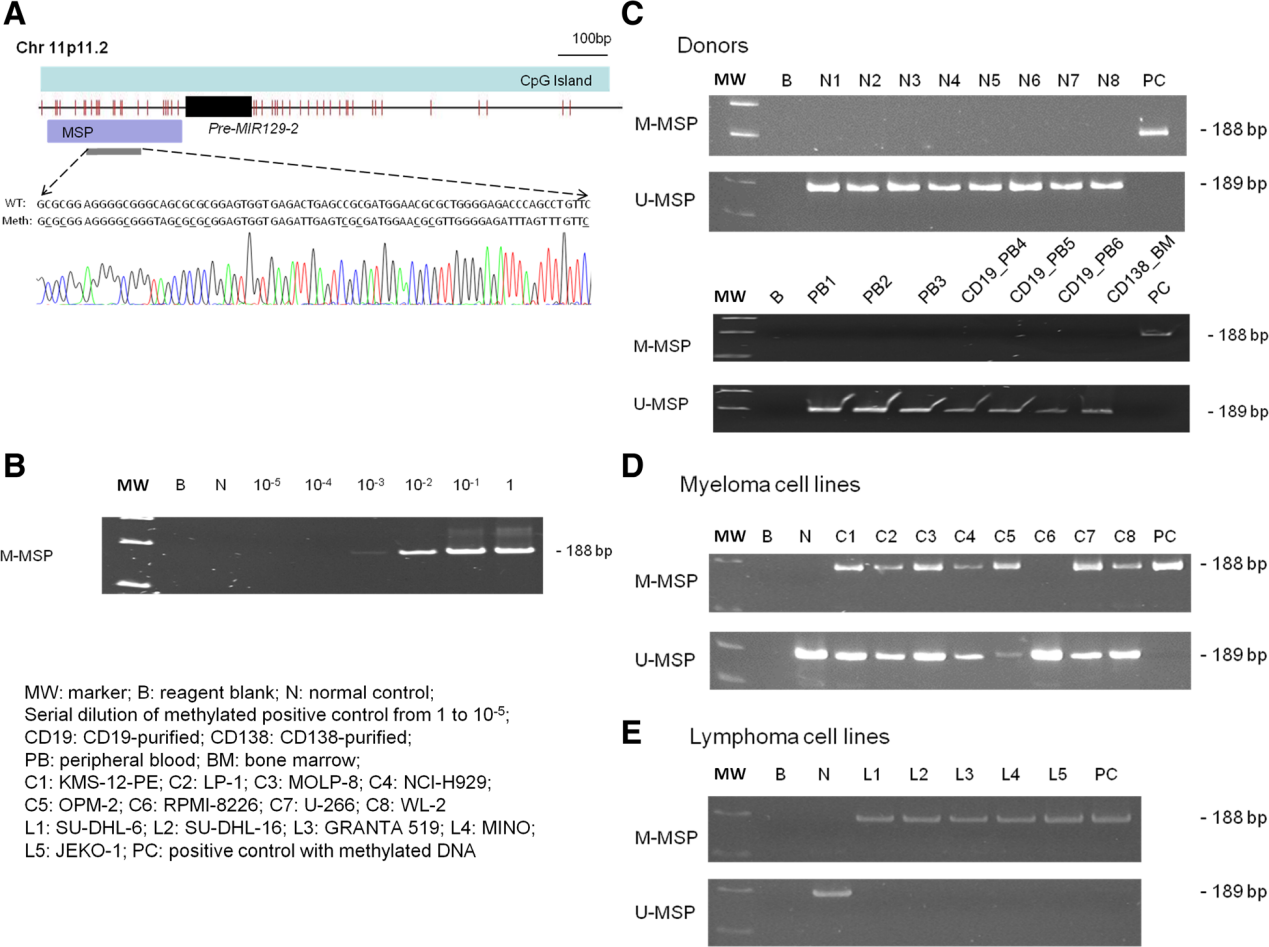
**Methylation of *MIR129-2.*** (**A**) Schematic diagram showing the distribution of CpG dinucleotides (solid vertical lines) along precursor *MIR129-2 *and its upstream promoter region. Region of MSP amplification was indicated by the text box marked as “MSP”. Sequence analysis of the *MIR129-2 *M-MSP product from bisulfite-treated methylated control DNA showed that the cytosine [C] residues of CpG dinucleotides were methylated and remained unchanged, whereas all the other C residues were unmethylated and were converted to thymidine [T], indicating complete bisulfite conversion and specificity of MSP. (**B**) Sensitivity of the methylated-MSP for the *MIR129-2*. (**C**) M- & U-MSP of 15 healthy donor controls showing no *MIR129-2 *methylation. (**D**) All myeloma cell lines, except RPMI-8226, were partially methylated, while RPMI-8226 was completely unmethylated for *MIR129-2*. (**E**) All five lymphoma cell lines were completely methylated for *MIR129-2*.

Direct sequencing analysis of M-MSP products of a methylated positive control showed expected conversion of unmethylated cytosine to uracil (turned into thymidine after PCR) while leaving methylated cytosine unchanged, which indicated complete bisulfite conversion and MSP specificity (Figure 1A). Sensitivity of the *MIR129-2* M-MSP was one in 10^3^ (Figure 1B). None of the 15 healthy donor samples showed aberrant *MIR129-2* methylation (Figure 1C). On the other hand, 7 of 8 MM cell lines showed partial *MIR129-2* methylation (Figure 1D). Moreover, all of the 5 lymphoma cell lines showed complete *MIR129-2* methylation (Figure 1E). Quantitative bisulfite pyrosequencing confirmed the methylation statuses (MM, MU, UU) of the cell lines detected by MSP (Additional file 1: Figure S1A and B). Furthermore, of these completely or partially methylated cell lines, complete methylation of the *MIR129-2* was associated with a trend of lower *MIR129* expression than those with partial methylation (Additional file 1: Figure S2).

### Methylation-specific PCR: *MIR129-2* methylation in primary samples at diagnosis

**Figure 2:**
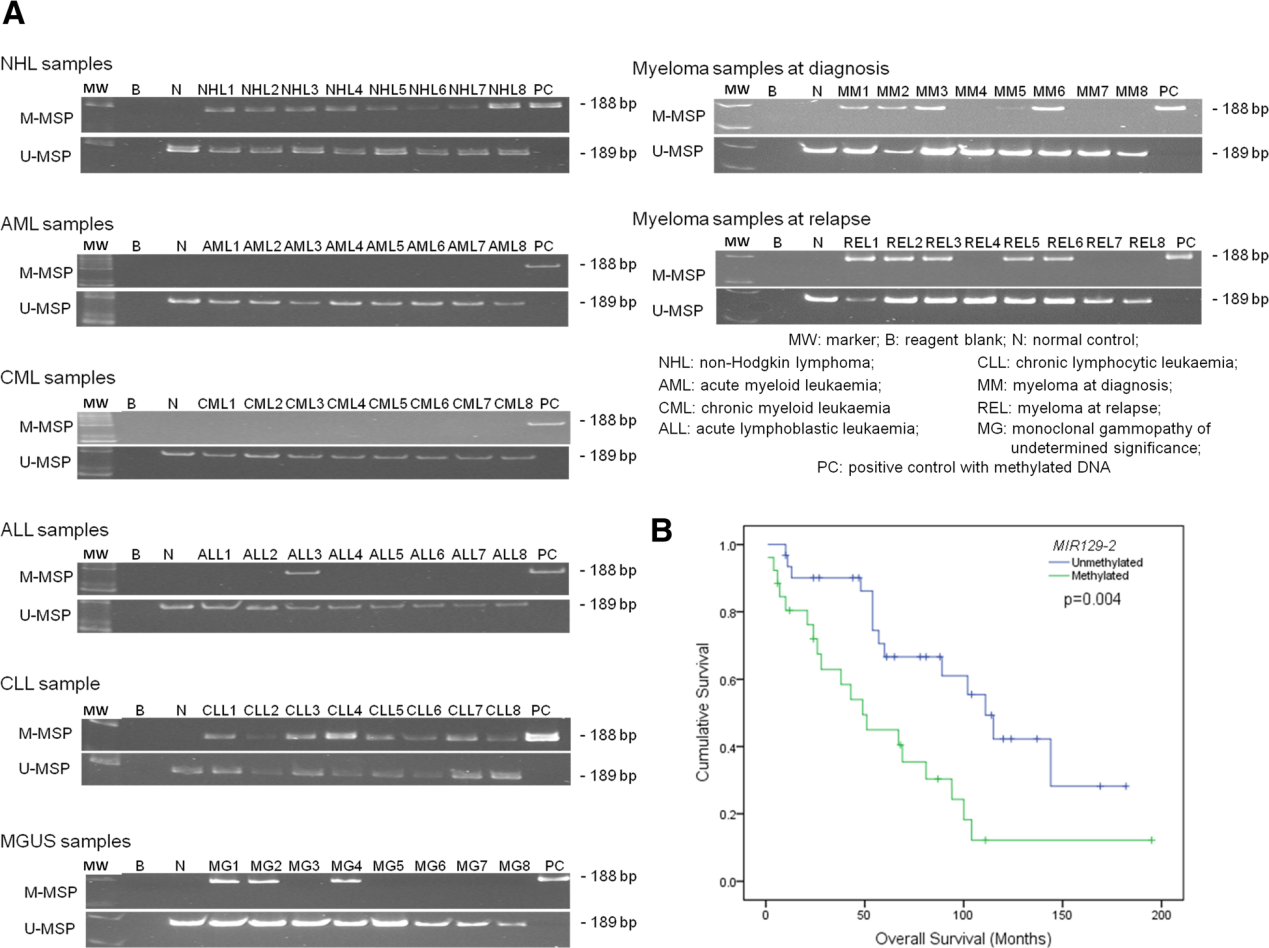
**Methylation of *MIR129-2 *in primary samples of hematological malignancies. **(**A**) M- & U-MSP analysis of *MIR129-2 *in primary samples of AML, CML, ALL, CLL, NHL, myeloma at diagnosis, and MGUS and myeloma at relapse. (**B**) Kaplan Meier survival function of CLL patients with and without *MIR129-2* methylation.

There was no *MIR129-2* methylation detected in any of the AML and CML patients (Figure 2A). In ALL, *MIR129-2* methylation was detected in only 1 (5%) of 20 patients. In CLL, *MIR129-2* methylation occurred in 28 (45.9%) patients (Figure 2A). *MIR129-2* methylation was not correlated with mean or median hemoglobin level, lymphocyte and platelet counts. Moreover, there was no correlation between *MIR129-2* methylation and age, gender, Rai stage (≥stage 2), or high-risk karyotypic aberrations. In 50 CLL patients with concomitant data on *MIR34A*, *MIR124-1*, *MIR196B* and *MIR203* methylation, there was no association with *MIR129-2* methylation with methylation of these microRNAs (data not shown). On the other hand, the median survivals were significantly inferior in patients with *MIR129-2* methylation than those without (49 *versus* 111 months, p=0.004; Figure 2B).

**Figure 3:**
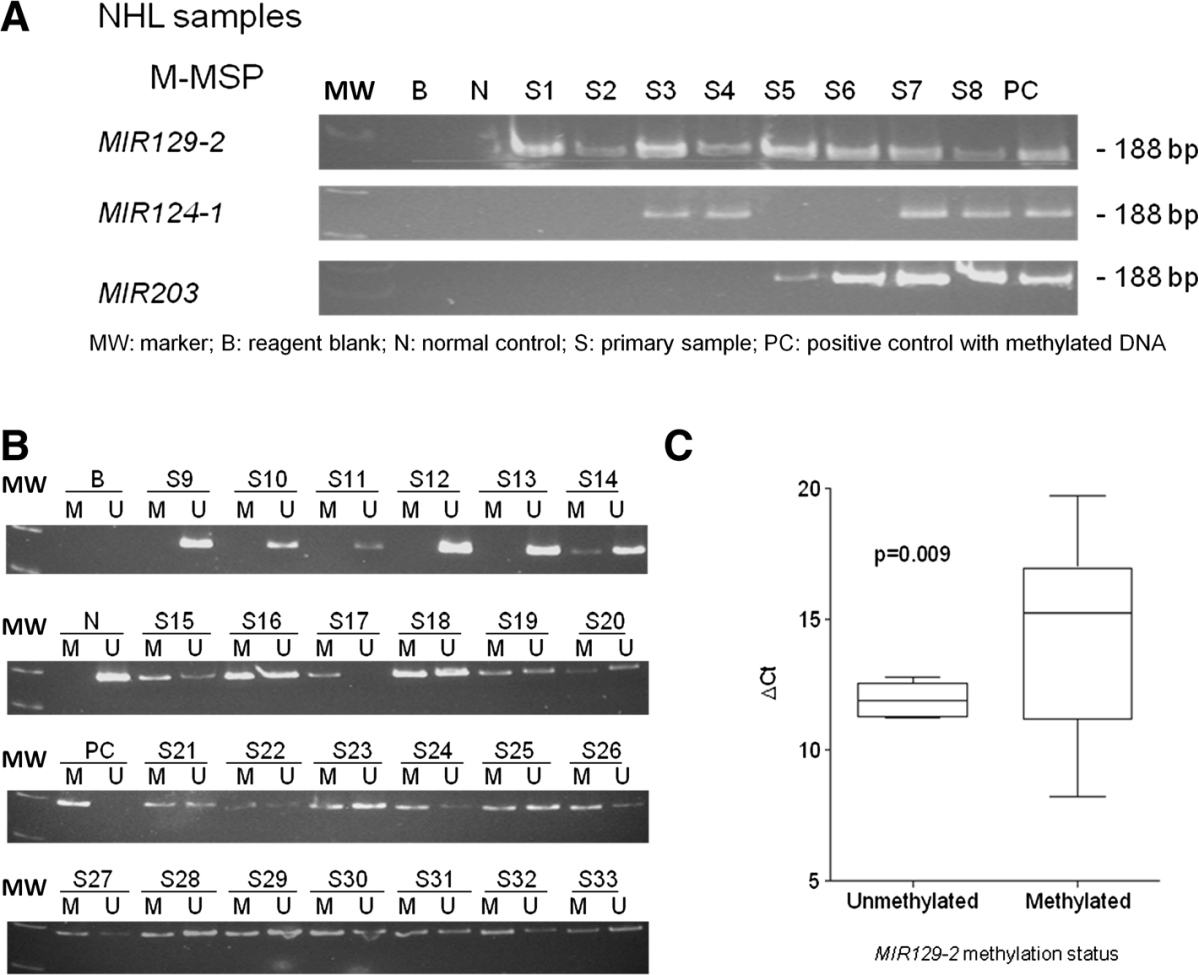
**Methylation of *MIR129-2 *and expression of *MIR129 *in primary lymphoma samples. **(**A**) Correlations of *MIR129-2, MIR203 *and *MIR124-1 *methylation in samples as shown by M-MSP analysis from 8 representative lymphoma samples. (**B**) M- & U-MSP analysis of *MIR129-2 *methylation and (**C**) stem-loop RT-qPCR analysis of *MIR129 *expression in 25 primary NHL samples with matched DNA and RNA. Box-and-whisker plot showed the *MIR129 *expression (ΔC_t_: C_t *MIR129 *_– C_t *SNORD48*_) in methylated and unmethylated primary NHL samples. The box indicated the 25th and 75th percentile and the whiskers represent the range. The horizontal line indicated the median. P-value was compared by two-sided Student *t*-test.

Amongst NHL samples, *MIR129-2* was methylated in 41 (60.3%) cases, including four NK/T-cell lymphoma (50.0%), 31 B-cell lymphomas (68.9%), and six T-cell lymphomas (40.0%). Methylation of *MIR129-2* was not correlated with age, gender, extranodal disease or Ann Arbor staging. On the other hand, *MIR129-2* methylation was associated with methylation of *MIR124-1* (N=68; p<0.001), *MIR203* (N=43; p<0.001) but not that of *MIR34A* or *MIR196B* (Figure 3A). Finally, in 25 primary lymphoma samples (follicular lymphoma, N=12; diffuse large B-cell lymphoma, N=13) with both DNA and RNA available, 20 samples displayed methylated MSP signals and 5 were completely unmethylated (Figure 3B), which were verified by quantitative pyrosequencing in selected cases (Additional file 1: Figure S3A and B). Importantly, samples with methylation of *MIR129-2* was associated with a lower level of mature *MIR129* expression, and hence a higher ΔC_t_ (C_t__*MIR129*_ - C_t__*SNORD48*_) than those completely unmethylated samples (p=0.009) (Figure 3C).

In MM, *MIR129-2* was methylated in 47 patients (49.5%) at diagnosis and 12 patients (41.4%) at relapse (p=0.590). Methylation of *MIR129-2* was more frequent in IgD immunoglobulin isotype, occurring in 3 (100%) of IgD, 34 (59.5%) of IgG, 8 (34.8%) of IgA, and 2 (18.2%) of light chain but none of non-secretary MM (p=0.013). However, there was no association of *MIR129-2* methylation with gender, ISS, median OS, or methylation of *MIR34A*, *MIR124-1*, *MIR196B* or *MIR203*. Interestingly, *MIR129-2* methylation was only detected in 11 (27.5%) patients with MGUS. Therefore, *MIR129-2* methylation was more frequent in MM at diagnosis than patients with MGUS (p=0.023).

### 5-azadC treatment and *MIR129* overexpression in lymphoma cell lines

**Figure 4:**
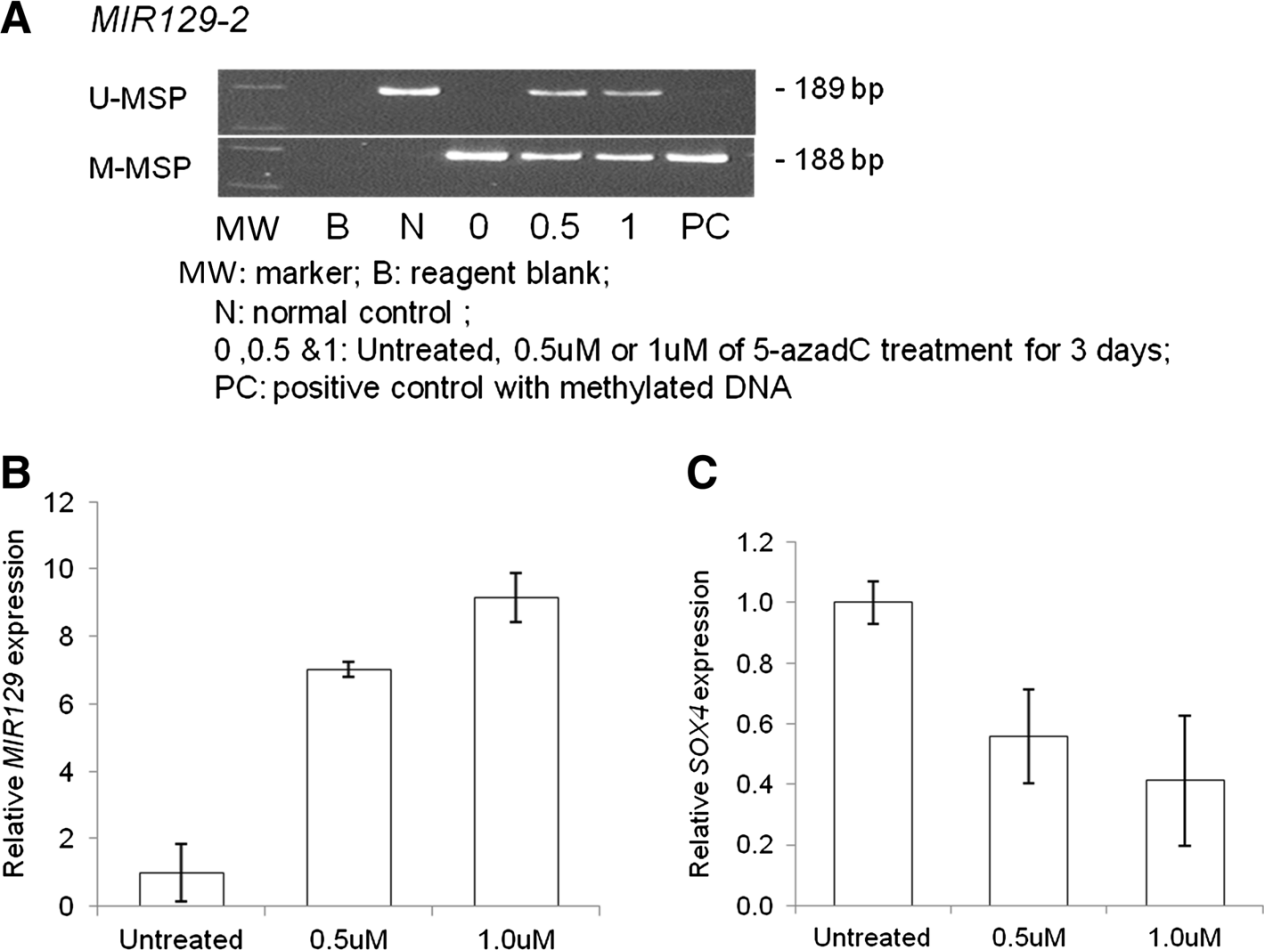
**5-azadC treatment induced DNA demethylation and re-expression of *MIR129 *leading to re-pression of *SOX4.*** (**A**) M- & U-MSP of JEKO-1 lymphoma cells upon 5-azadC treatment. (**B**) RT-qPCR analysis of the mature *MIR129 *expression upon treatment. (**C**) RT-qPCR analysis of the *SOX4 *expression upon treatment.

**Figure 5:**
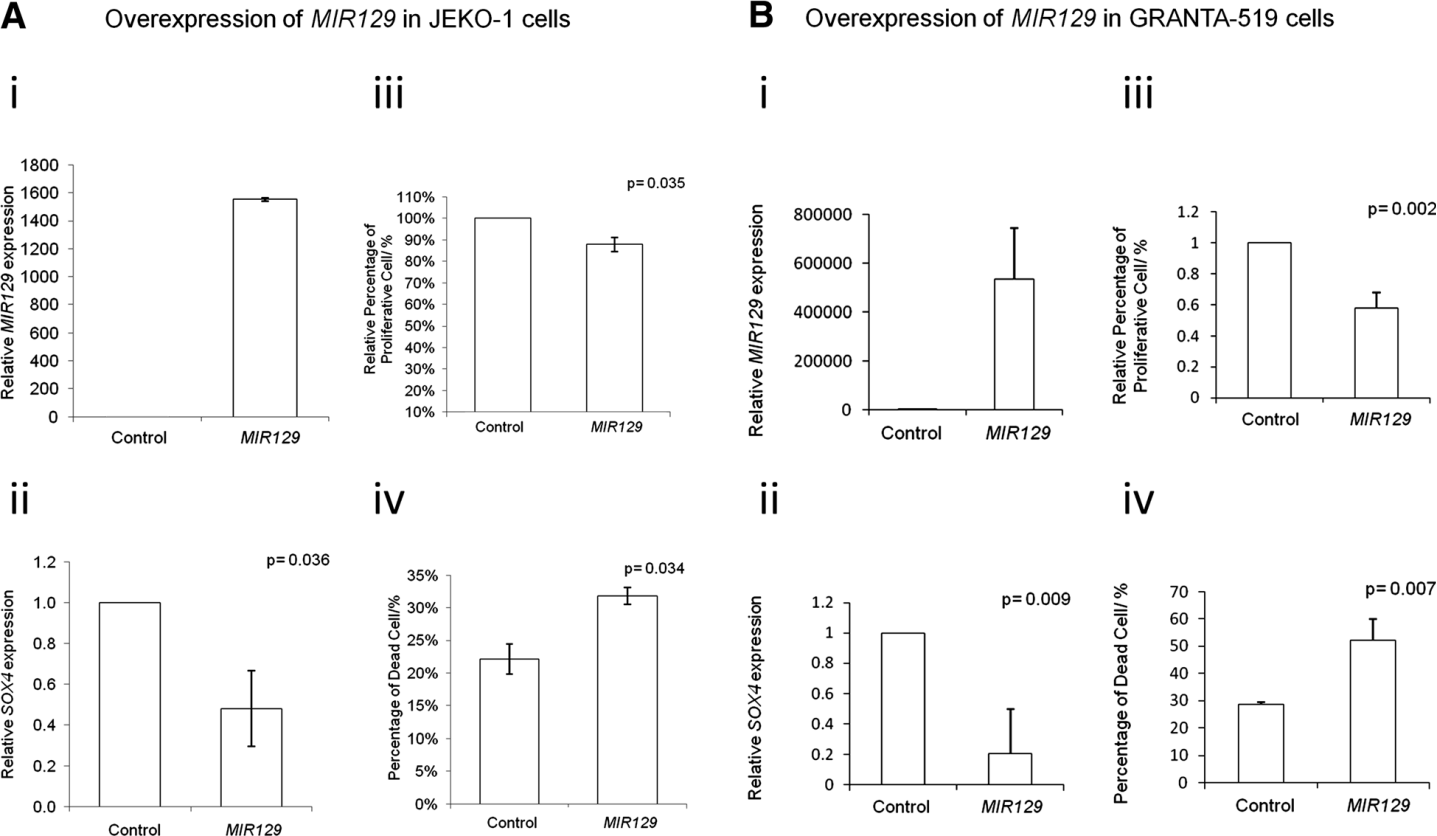
**Ectopic re-expression of *MIR129 *inhibit cell proliferation and elicited cell death in JEKO-1 and GRANTA-519 cells. **(**A**) JEKO-1. (**B**) GRANTA-519. (**i**) RT-qPCR analysis of mature *MIR129* after 48hrs of transfection of lymphoma cells. (**ii**) RT-qPCR analysis of the *SOX4 *expression upon *MIR129 *overexpression. (**iii**) Cell proliferation of lymphoma cells in response to overexpression of *MIR129 *measured by MTT assay. (**iv**) Percentage of dead cells in response to *MIR129 *overexpression measured by trypan blue exclusion method. Error bars represent standard deviation.

To investigate if the *MIR129-2* methylation might lead to low *MIR129* expression, JEKO-1 with homozygously methylated *MIR129-2* was treated with different concentrations of 5-azadC for 3 days and tested for MSP and stem-loop RT-qPCR. On treatment with 5-azadC, *MIR129-2* was demethylated (Figure 4A; Additional file 1: Figure S4), with a corresponding increase in *MIR129* expression (Figure 4B) and downregulation of *SOX4* mRNA (Figure 4C), which has been shown to be a direct target of *MIR129*. To validate the tumor suppressive effect of *MIR129*, *MIR129* was overexpressed in JEKO-1 cells (Figure 5A). Upregulation of *MIR129* led to *SOX4* downregulation (p=0.036, Figure 5Aii). Furthermore, *MIR129* expression resulted in reduction of cellular proliferation as measured by MTT assay (p=0.018, Figure 5Aiii) and an increase of dead cells as measured by trypan blue exclusion assay (p=0.017, Figure 5Aiv). The tumor suppressive effect of *MIR129* in the inhibition of cell proliferation and enhancement of cell death was further demonstrated in GRANTA-519 cells, which is also completely methylated for *MIR129-2* (Figure 5B).

## Discussion

In this study, we demonstrated that *MIR129-2* was hypermethylated in NHL and MM cell lines but not in normal blood or mononuclear cells, illustrating a methylation pattern similar to other epigenetically silenced tumor suppressor microRNAs, such as *MIR34A*, *MIR34B/C*, *MIR124*, and *MIR203*, in hematological cancers [13-17]. This is in contrast to some methylated microRNAs, such as *MIR127* and *MIR373*, which show a tissue-specific methylation pattern, with methylation occurring in both tumor cells and their normal counterparts [18]. Moreover, methylation leading to reversible gene silencing was illustrated here with re-expression of *MIR129* upon hypomethylation of *MIR129-2*. Furthermore, overexpression of *MIR129* led to decreased cell proliferation with increased cell death. These results were consistent with a tumor suppressor role of *MIR129* in lymphoma cells, similar to its effects on other epithelial cancers [9-11,19]. In particular, *SOX4*, a known target of *MIR129*, facilitates differentiation of lymphocytes, and has been shown upregulated in various human cancers [20]. Indeed, herein, downregulation of *SOX4* was shown associated with upregulation of *MIR129* upon either hypomethylating treatment or overexpression in GRANTA-519 and JEKO-1 lymphoma cell lines. Taken together, the findings indicate that hypermethylation of *MIR129-2* led to reversible inactivation of tumor suppressive *MIR129* in hematological cancers. Lastly, in cell lines which showed complete methylation of *MIR129-2*, there is no deletion of the *MIR129-2* locus, i.e. chromosome 11p11 [21], and hence complete methylation in these cells suggests biallelic *MIR129-2* methylation.

Secondly, we found that *MIR129-2* methylation was frequent and appeared to be associated with poor survival in CLL patients, which warrants future prospective studies with larger number of patients. In CLL, apart from *ZAP-70* gene hypermethylation being a favourable prognostic marker, there is little information on the role of DNA methylation in the pathogenesis and clinical outcome of the disease [22-26]. Furthermore, understanding of the prognostic value of microRNA and microRNA methylation in CLL remains preliminary [14-16,27-29]. Hence, our observation of *MIR129-2* methylation adversely impacting on survival in CLL is a novel finding. In order to establish the prognostic significance of *MIR129-2* methylation in CLL, a multivariate analysis together with Rai stage, lymphocyte counts and high-risk karyotype is required. However, the small number of patients in this cohort precluded a multivariate analysis.

In NHL, in contrast to *MIR34A*, *MIR124-1*, and *MIR203*, which were frequently methylated in NK- or B-cell lymphoma, *MIR129-2* methylation was frequent but comparable among B-, T- or NK-cell lymphomas. However, an interesting observation was that methylation of *MIR129-2,* which is localized to chromosome 11p11, was associated with methylation of *MIR124-1* (localized to 8p23) and *MIR203* (localized to 14q32). As *MIR124-1* targets *CDK6* mRNA and *MIR203* targets *ABL* and *CREB* mRNAs, the strong association of methylation of these microRNAs suggested collaboration of silencing of multiple microRNAs for oncogenesis [14,16]. Moreover, in lymphoma samples, in which both DNA and RNA were available, significantly lower expression of *MIR129* was demonstrated in primary lymphoma samples with *MIR129-2* methylation than those without, further testifying the association of microRNA silencing with microRNA hypermethylation.

In MM, *MIR129-2* methylation was more frequent in MM patients at diagnosis or relapse/progression than patients with MGUS, and hence might be an important event implicated in transformation of MGUS to symptomatic MM. Despite that these samples were not CD138-sorted, the mean and median of plasma cell percentage of these MGUS samples were 4.78 and 5 respectively, and hence well within the limit of detection by the M-MSP. However, MSP performed on CD138-sorted plasma cells would be ideal, and hence the current finding warrants further studies using CD138-sorted samples. On the other hand, there was no impact of *MIR129-2* methylation on OS. However, this cohort of patients was heterogeneously-treated, and hence the prognostic impact of *MIR129-2* methylation remains to be verified in a cohort of uniformly-treated patients.

## Conclusions

In summary, *MIR129* is a putative tumor suppressive microRNA, and methylated in a tumor-specific manner, leading to reversible microRNA silencing. *MIR129-2* methylation was frequent in lymphoid but uncommon in myeloid neoplasms. In CLL, *MIR129-2* methylation adversely impacted on survival. In NHL, *MIR129-2* methylation was associated with methylation of other tumor suppressor microRNAs. In MM, *MIR129-2* methylation was probably associated with progression from MGUS to symptomatic MM. Therefore, *MIR129-2* methylation is important in the pathogenesis, disease progression and prognostication in lymphoid neoplasms. The implication of *MIR129-2* methylation with methylation of other tumor suppressive microRNAs in lymphomas warrants further study.

## Methods

### Patient samples

Diagnostic bone marrow or tissue samples were obtained in 20 ALL, 20 AML, 11 CML in chronic phase, 61 CLL, 68 NHL, 40 MGUS, 95 MM at diagnosis, and 29 MM at relapse/progression. Patient demographics were listed in Table 1.

**Table 1:** Patient demographics


ALL (N = 20)	Gender (M/F)	11/9
	Median age (range)	35 (13–62) years
	MIC type (C/PB/EPB/T)	6/10/1/3
AML (N = 20)	Gender (M/F)	9/11
	Median age (range)	41.5 (20–72) years
	FAB type (M1/M2/M4/M5)	3/14/2/1
CLL (N = 61)	Gender (M/F)	44/17
	Median age (range)*	65 (37–91) years
	Rai stage (<2/≥2)*	37/20
	Median lymphocyte count (range)*	18.5 (10–236) × 10^9^/L
	High-risk [del(17p)/trisomy 12]‡	1/7
	Low-risk [del(13)/normal karyotype/ other karyotype abnormalities]‡	7/16/6
CML (N = 11)	Gender (M/F)	7/4
	Median age (range)	41 (22–87) years
	Chronic phase	11
MM (N = 95)	Gender (M/F)	37/58
	Median age (range)	62 (29–91) years
	Ig type (G/A/D/LC/NS)	57/23/3/11/1
	ISS stage (I/II/III)†	16/36/27
NHL (N = 68)	Gender (M/F)	38/30
	Median age (range)	60.5 (17–92)
	Ann Arbor stage (I/II/III/IV)^	3/4/4/18
	Type (ALCL/ AITL/ PTCL,NOS/ NK-T/ FL/ MZL/ MCL/ DLBCL)	2/ 4/ 9/ 8/ 21/ 7/ 2/ 15

Keys: *, from 59 patients with clinical data; ‡, from 37 patients with cytogenetic data; †, from 79 patients with sufficient data; ^, from 29 patients with Ann Arbor stage data; A, IgA type; AITL, angioimmunoblastic T-cell lymphoma; ALCL, anaplastic large-cell lymphoma; C, common ALL; D, IgD type; DLBCL, diffuse large B-cell lymphoma; EBP, early B precursor; FAB, French-American-British; FL, follicular lymphoma of grade ≤ 2; G, IgG type; ISS, International Staging System for myeloma; LC, light chain myeloma; MCL, mantle cell lymphoma; MIC, morphologic-immunophentypic-classification; MZL, marginal zone lymphoma; NK-T, NK/T-cell lymphoma (nasal or extranasal type); NS, non-secretary myeloma; PB, precursor B ALL; PTCL,NOS, peripheral T-cell lymphoma, not otherwise specified; T, precursor T ALL.

Diagnosis of leukemia and lymphoma were made according to the French-American-British Classification and WHO Classification of Tumors respectively [30-33].

In the CLL group, median overall survival (OS) was 81 months for the whole group, and 102 months in those with limited, and 54 months in those with advanced Rai stage (p=0.009). Median OS of CLL patient with low/standard-risk and high-risk karyotypes were 111 months and 21 months (p<0.001).

In the NHL group, of 36 patients with data available at clinical presentation, 23 had nodal and 13 had extranodal involvement. Correlation between microRNA methylation and expression was studied in 25 primary lymphoma samples (follicular lymphoma, N=12; diffuse large B-cell lymphoma, N=13), in which both DNA and RNA were available.

The diagnosis of MGUS and MM was based on standard criteria [34]. Complete staging work-up included bone marrow examination, skeletal survey, serum and urine protein electrophoresis, and serum immunoglobulin (IgG, IgA, and IgM) levels. In this cohort, the median OS was 44 months, and projected 10-year OS was 19.9%. The median OS were 83 months, 60 months and 23 months in those with ISS I, II and III disease respectively (p<0.001). Definitions of relapse and disease progression followed the criteria of European Group for Blood and Marrow Transplantation Registry [35]. Briefly, “relapse” from complete remission (CR) was defined as the reappearance of the same paraprotein detected by serum/urine protein electrophoresis, appearance of new bone lesion or extramedullary plasmacytoma, or unexplained hypercalcaemia. The definition of “disease progression” from plateau phase/stable disease was the same as the definition of relapse except that “a >25% increase in paraprotein level” replaced “reappearance of the same paraprotein”. The study has been approved by Institutional Review Board of Queen Mary Hospital, and written informed consent was obtained from the patient for publication of this report and any accompanying data or images.

### Cell culture

MM cell lines LP-1 & RPMI-8226 were kindly provided by Dr Robert Orlowski (Department of Lymphoma/Myeloma, Division of Cancer Medicine, The University of Texas MD Anderson Cancer Center, Houston, TX, USA) and WL-2 by Prof. Andrew Zannettino (Myeloma Research Programme, The University of Adelaide, Australia). NCI-H929 was purchased from American Type Culture Collection (Manassas, VA, USA). Other MM (KMS-12-PE, MOLP-8, OPM-2 and U-266) and lymphoma (SU-DHL-6, SU-DHL-16, GRANTA-519, MINO & JEKO-1) cell lines were purchased from Deutsche Sammlung von Mikroorganismen und Zellkulturen (DSMZ) (Braunschweig, Germany). Cell cultures were maintained in RPMI-1640 (IMDM for LP-1), supplemented with 10% (15% for lymphoma cell lines) fetal bovine serum, 50 U/ml of penicillin and 50 ug/ml streptomycin in a humidified atmosphere of 5% CO2 at 37°C. All cell culture reagents were purchased from Invitrogen (Carlsbad, CA, USA).

### DNA and RNA extractions

DNA was extracted from primary samples, 8 MM cell lines (KMS-12-PE, LP-1, MOLP-8, NCI-H929, OPM-2, RPMI-8226, U-266 and WL-2) and 5 lymphoma cell lines (SU-DHL-6, SU-DHL-16, GRANTA-519, MINO and JEKO-1), using QIAamp DNA Blood Mini (Qiagen, Hilden, Germany). Total RNA were harvested using mirVana™ miRNA Isolation Kit (Ambion Austin, TX, USA).

### Methylation-specific polymerase chain reaction (MSP)

DNA samples were treated to convert unmethylated cytosine to uracil by EpiTect Bisulfite Kit (Qiagen, Hilden, Germany). Primers and conditions for methylated-MSP (M-MSP) and unmethylated-MSP (U-MSP) of *MIR129-2* were listed in Table 2. Primers and conditions of MSP for *MIR124-1* and *MIR203* were previously described [14,16]. Mononuclear cell DNA from 15 healthy donors [8 bone marrow, 3 peripheral blood, 3 CD19-sorted peripheral blood, and 1 CD138-sorted bone marrow (AllCells, CA, USA)] were used as negative control, and an enzymatically methylated control DNA purchased from CpGenome Universal Methylated DNA (Chemicon/Millipore, Billerica, MA, USA) was used as positive control in all the experiments. Amplified products were then visualized by 6% non-denaturing polyacrylamide gel stained by ethidium bromide.

**Table 2:** Primer sequences and reaction conditions

**Gene**	**Forward primer (5’ – 3’)**	**Reverse primer (5’ – 3’)**	**Product size (bp)**	**Tm/cycles**
(I) Methylation-specific polymerase chain reaction (MSP)				
*MIR129-2*				
M-MSP	GAGTTGGGGGATCGCGGAC	ATATACCGACTTCTTCGATTCGCCG	188	59°C/ 35
U-MSP	GAGTTGGGGGATTGTGGAT	AATATACCAACTTCTTCAATTCACCA	189	55°C/ 35
(II) Reverse transcription-polymerase chain reaction (RT-PCR)				
*SOX4*	GCTGGAAGCTGCTCAAAGAC	ACCGACCTTGTCTCCCTTCT	167	60°C/ 40
*GAPDH*	ACCACAGTCCATGCCATCACT	TCCACCACCCTGTTGCTGTA	452	60°C/ 40

Keys: M-MSP, MSP for the methylated allele; Tm, annealing temperature; U-MSP, MSP for the unmethylated allele.

### Sensitivity of the M-MSP

To establish the sensitivity of the *MIR129-2* M-MSP, 1 μg of methylated control DNA was 10-fold serially diluted in buffer, bisulfite-treated and amplified with *MIR129-2* M-MSP primers.

### Quantitative bisulfite pyrosequencing

Bisulfite-treated DNA was used as template. Methylation-unbiased primer set was used to amplify the promoter region, which overlapped with the amplicon of the MSP. Forward: 5’-AGA GGG ATA GGA TAG GTA GG-3’; reverse: 5’-AAC CCT AAA ACC CAA CAA ACT AAA TCT-3’; condition: 2 mM/55°C/50X. A stretch DNA with 9–12 adjacent CpG dinucleotides was pyrosequenced by sequencing primer: 5’-GGT TTG GAG AAA TGG A-3’.

### Hypomethylating treatment

JEKO-1 was homozygously methylated for *MIR129-2*. Cells were seeded in six-well plates at a density of 1x10^6^ cells/ml and cultured with 0.5–1uM of 5-aza-2’-deoxycytidine (5-azadC) (Sigma–Aldrich) for 3 days.

### Quantitative real-time reverse transcription–PCR (RT-qPCR)

Short mature microRNA transcripts were quantified using stem-loop RT-qPCR which is a sensitive, specific and widely-used method designed for microRNA studies [36]. For *MIR129*, RT was performed using Taqman® MicroRNA RT Kit and Taqman® MicroRNA Assay Kit (ABI, Foster City, CA, USA), according to the manufacturer’s instructions. Total RNA was reverse transcribed in 1 mmol/l dNTPs, 50 U MultiScribe™ Reverse Transcriptase, 1× RT Buffer, 3·8 U RNase Inhibitor, and 1× stem-loop RT primer at following thermal cycling condition: 16°C for 30 min, 42°C for 30 min, and 85°C for 5 min. RT-qPCR of *MIR129* was performed using 1·33 μl of 1:15 diluted RT product in 1× Taqman® Universal PCR Master Mix, and 1× Taqman® Assay at 95°C for 10 min, followed by 40 cycles of 95°C for 15 s and 60°C for 1 min. *SNORD48* was used as reference for data analysis with the 2^-ΔΔCt^ method [37]. Conventional RT-qPCR was used for *SOX4* transcript, RT was performed using QuantiTect Reverse Transcription Kit (Qiagen), according to the manufacturer’s instructions. RT-qPCR was performed by iQ SYBR Green Supermix (Bio-Rad), using *GAPDH* as endogenous control for data analysis with the 2^-ΔΔCt^ method [37]. Primers for detecting *SOX4* and *GAPDH* were summarized in Table 2.

### *MIR129* overexpression in JEKO-1 cells

Cells at log phase were transfected with 150nM of either negative control mimic or *MIR129* oligo mimic (Ambion) at a density of 10^6^ cell/mL using X-tremeGENE siRNA transfection reagent (Roche), according to the manufacturer’s instructions.

### MTT assay

Cell proliferation was determined by colorimetric quantification of purple formazan formed from the reduction of yellow tetrazolium MTT (3-(4, 5-dimethylthiazolyl-2)-2, 5-diphenyltetrazolium bromide) by proliferating cells. Briefly, cells were seeded in a 96-well microtitre plate at 5 × 10^5^ /well in 100 μl of medium. At the assay time point, each well was added 10 μl of 5 mg/ml MTT reagent (Sigma-Aldrich), followed by 6-hour incubation, after which 100 μl of DMSO was added. The absorbance reading at 550 nm with reference to 650 nm was recorded. Relative abundances of proliferative viable cells from three independent experiments were calculated.

### Trypan blue exclusion assay

Dead cells were visualized by trypan blue staining and five random microscopic fields were counted for each sample. Dead cells (%) = (total number of dead cells per microscopic field/ total number of cells per microscopic field) X 100. Percentages of dead cells from three independent experiments were calculated.

### Statistical analysis

Correlation between *MIR129-2* methylation with continuous (mean age) and categorical variables (gender, histological subtypes, lineage [B, T or NK/T] and nodal/extranodal presentation) were studied in these 68 patients by Student’s *t*-test and Chi-square test (or Fisher Exact test) respectively. Overall survival (OS) was measured from the date of diagnosis to the date of last follow‐up or death. Survival was plotted by the Kaplan‐Meier method, and compared by the log‐rank test. Moreover, in 25 primary B-cell NHL samples in which both DNA and RNA were available, the mean expression of *MIR129* in methylated and unmethylated lymphoma was compared by the Student’s t-test. Association between *MIR129-2* methylation and other previously studied tumor suppressive microRNA methylation, including *MIR34A*, *MIR124-1*, *MIR203* and *MIR196B*[14-16], in MM, NHL and CLL patients were studied by Χ^2^ test. The mean results from triplicate experiments after *MIR129* transfection were compared by Student’s *t*-test. All p-values were 2-sided.

## Competing interests

The authors declare that they have no competing interests.

## Authors’ contributions

CSC designed the study. KYW, RLHY conducted the experiments. CCS, YLK, CYL, PKH, FC, RL helped in sample collection and clinical data retrieval. CSC, KYW, RLHY, DYJ helped in data analyses. All authors were involved in the writing and final approval of the manuscript.

## Supplementary Material

Additional file 1: Figure S1 Quantitative bisulfite pyrosequencing of MIR129-2. Pyrograms showing the methylation intensity on a stretch of 9 neighboring CpG dinucleotides of **(A)** Positive control with methylated DNA and normal control, and **(B) **Cell lines with defined MSP methylation statuese (MM, MU, and UU). **Figure S2. **Expression of MIR129 in cell lines completely or partially methylated for MIR129-2. Box-and-whisker plot showed complete methylation of MIR129-2 was associated with a lower level of MIR129 expression, and hence a higher ΔCt (Ct MIR129 - Ct SNORD48) than partial methylation. The box indicated the 25th and 75th percentile and the whiskers represent the range, or the 25th percentile minus 1.5 X interquartile range when skewed datum (*) exists. The horizontal line indicated the median. **Figure S3. **Quantitative bisulfite pyrosequencing of MIR129-2. Pyrograms showing the methylation intensity on a stretch of 9 neighboring CpG dinucleotides of **(A)** methylated primary NHL samples, and **(B) **unmethylated primary NHL samples. **Figure S4. **Quantitative bisulfite pyrosequencing of MIR129-2. Pyrograms showing the methylation intensity on a stretch of 9 neighboring CpG dinucleotides of JEKO-1 cells before and after 5-azadC treatment. Click here for file
