# Supplementary material for: Epigenetic inactivation of the MIR129-2 in hematological malignancies
Source: J Hematol Oncol. 2013 Feb 14;6:16. doi: 10.1186/1756-8722-6-16 (PMC3576298; doi:10.1186/1756-8722-6-16)
Supplement: Additional file 1: Figure S1 — Quantitative bisulfite pyrosequencing of MIR129-2. Pyrograms showing the methylation intensity on a stretch of 9 neighboring CpG dinucleotides of (A) Positive control with methylated DNA and normal control, and (B) Cell lines with defined MSP methylation statuese (MM, MU, and UU). Figure S2. Expression of MIR129 in cell lines completely or partially methylated for MIR129-2. Box-and-whisker plot showed complete methylation of MIR129-2 was associated with a lower level of MIR129 expression, and hence a higher ΔCt (Ct MIR129 - Ct SNORD48) than partial methylation. The box indicated the 25th and 75th percentile and the whiskers represent the range, or the 25th percentile minus 1.5 X interquartile range when skewed datum (*) exists. The horizontal line indicated the median. Figure S3. Quantitative bisulfite pyrosequencing of MIR129-2. Pyrograms showing the methylation intensity on a stretch of 9 neighboring CpG dinucleotides of (A) methylated primary NHL samples, and (B) unmethylated primary NHL samples. Figure S4. Quantitative bisulfite pyrosequencing of MIR129-2. Pyrograms showing the methylation intensity on a stretch of 9 neighboring CpG dinucleotides of JEKO-1 cells before and after 5-azadC treatment. [file 1756-8722-6-16-S1.pdf]

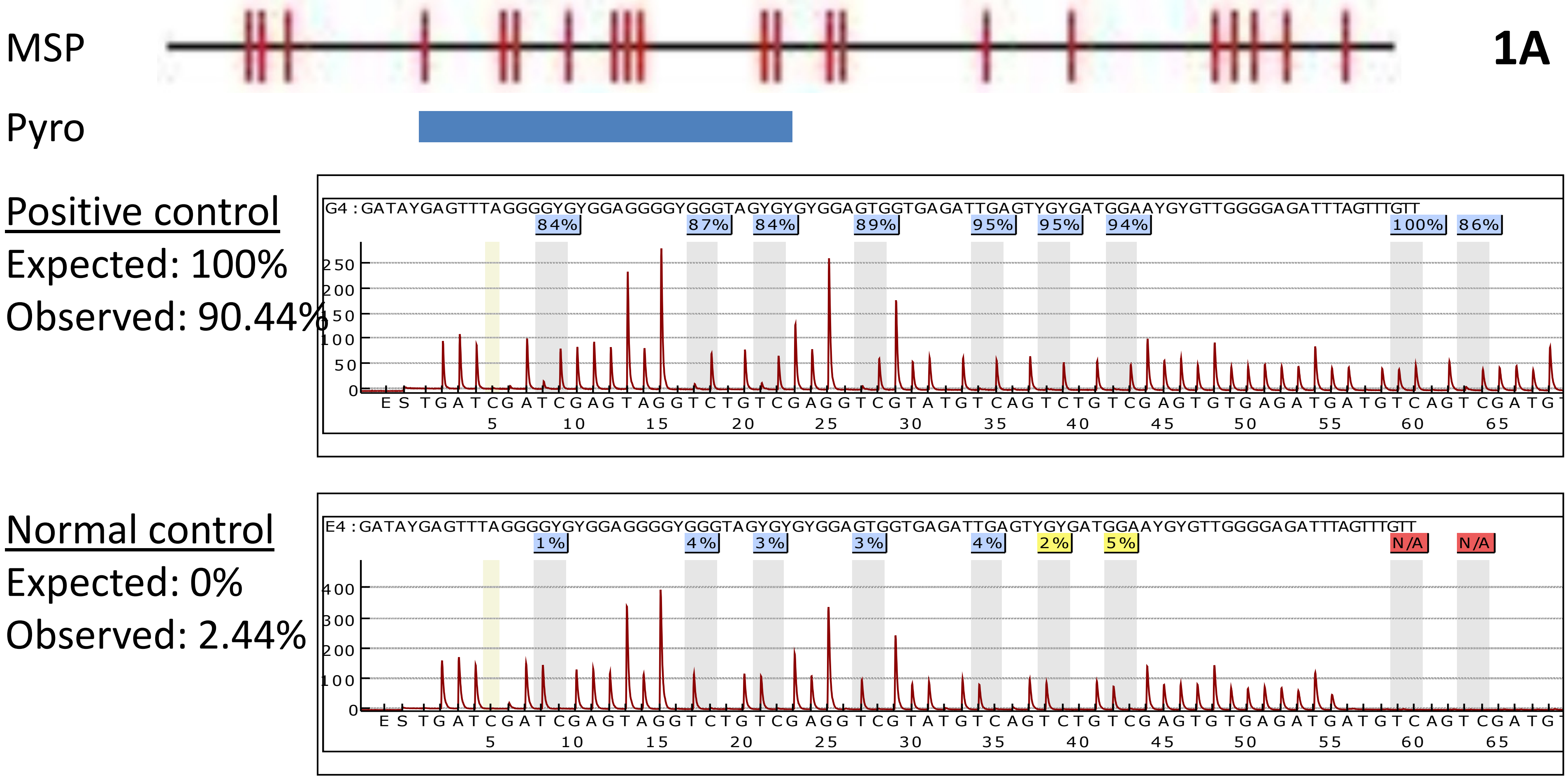

Additional Figure 1. Quantitative bisulfite pyrosequencing of MIR129-2. Pyrograms showing the methylation intensity on a stretch of 9 neighboring CpG dinucleotides of **(A)** Positive control with methylated DNA and normal control, and **(B)** Cell lines with defined MSP methylation statuese (MM, MU, and UU).

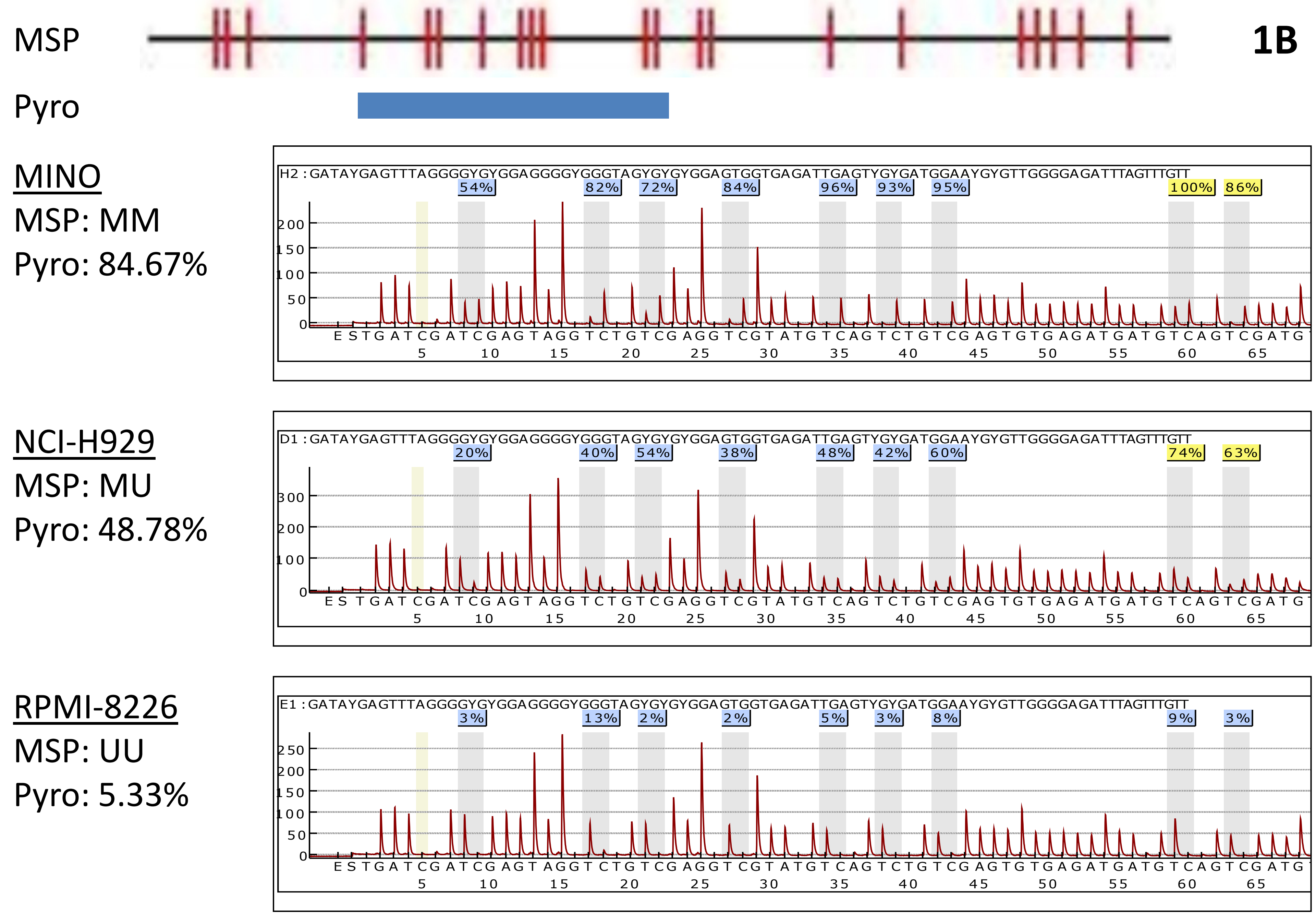
1B
MSP
Pyro
MINO
MSP: MM
Pyro: 84.67%
H2 : GATAYGAGTTTAGGGGYGYGGAGGGGYGGGTAGYGYGYGGAGTGGTGAGATTGAGTYGYGATGGAAYGYGTTGGGGAGATTTAGTTTGTT
54% 82% 72% 84% 96% 93% 95% 100% 86%
E S T G A T C G A T C G A G T A G G T C T G T C G A G G T C G T A T G T C A G T C T G T C G A G T G T G A G A T G A T G T C A G T C G A T G
NCI-H929
MSP: MU
Pyro: 48.78%
D1 : GATAYGAGTTTAGGGGYGYGGAGGGGYGGGTAGYGYGYGGAGTGGTGAGATTGAGTYGYGATGGAAYGYGTTGGGGAGATTTAGTTTGTT
20% 40% 54% 38% 48% 42% 60% 74% 63%
RPMI-8226
MSP: UU
Pyro: 5.33%
E1 : GATAYGAGTTTAGGGGYGYGGAGGGGYGGGTAGYGYGYGGAGTGGTGAGATTGAGTYGYGATGGAAYGYGTTGGGGAGATTTAGTTTGTT
3% 13% 2% 2% 5% 3% 8% 9% 3%

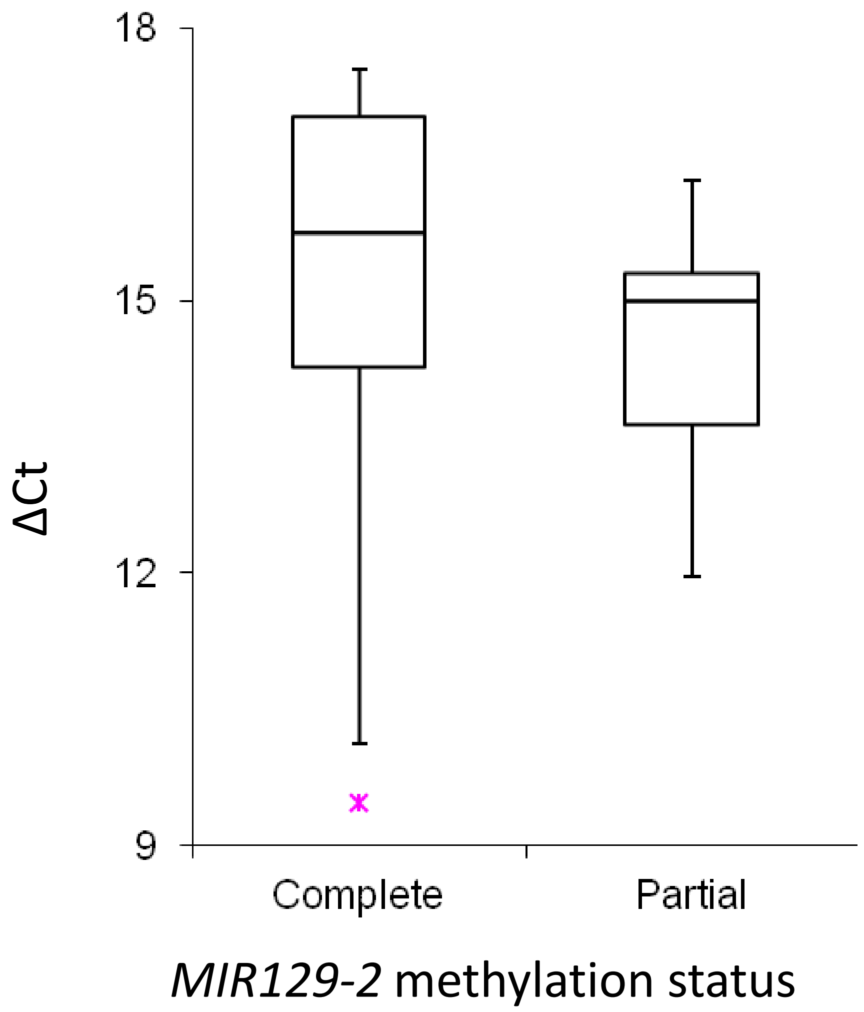

Additional Figure 2. Expression of MIR129 in cell lines completely or partially methylated for MIR129-2. Box-and-whisker plot showed complete methylation of MIR129-2 was associated with a lower level of MIR129 expression, and hence a higher ΔCt (Ct MIR129 - Ct SNORD48) than partial methylation. The box indicated the 25th and 75th percentile and the whiskers represent the range, or the 25th percentile minus 1.5 X interquartile range when skewed datum (*) exists. The horizontal line indicated the median.

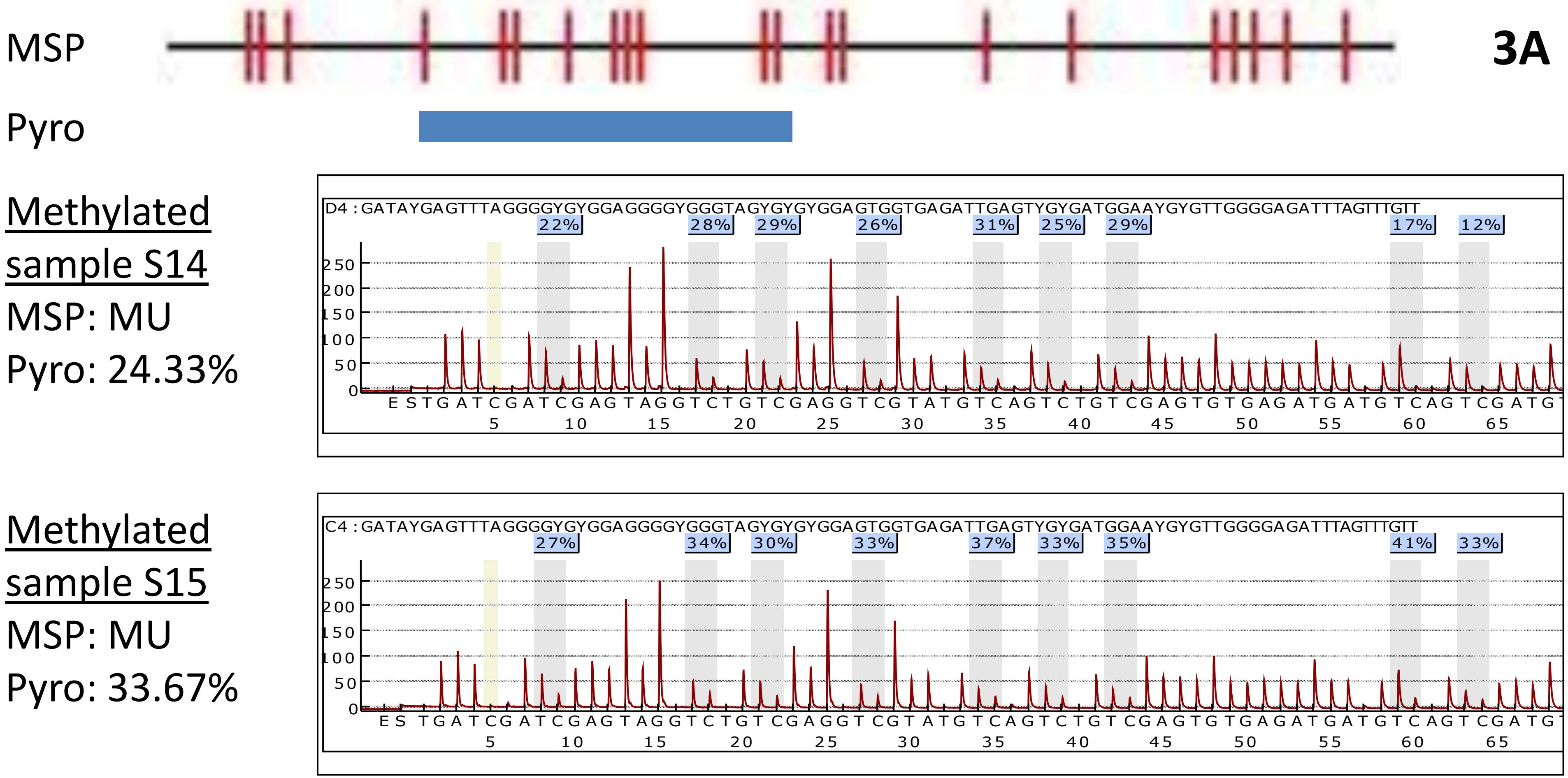

Additional Figure 3. Quantitative bisulfite pyrosequencing of MIR129-2. Pyrograms showing the methylation intensity on a stretch of 9 neighboring CpG dinucleotides of **(A)** methylated primary NHL samples, and **(B)** unmethylated primary NHL samples.

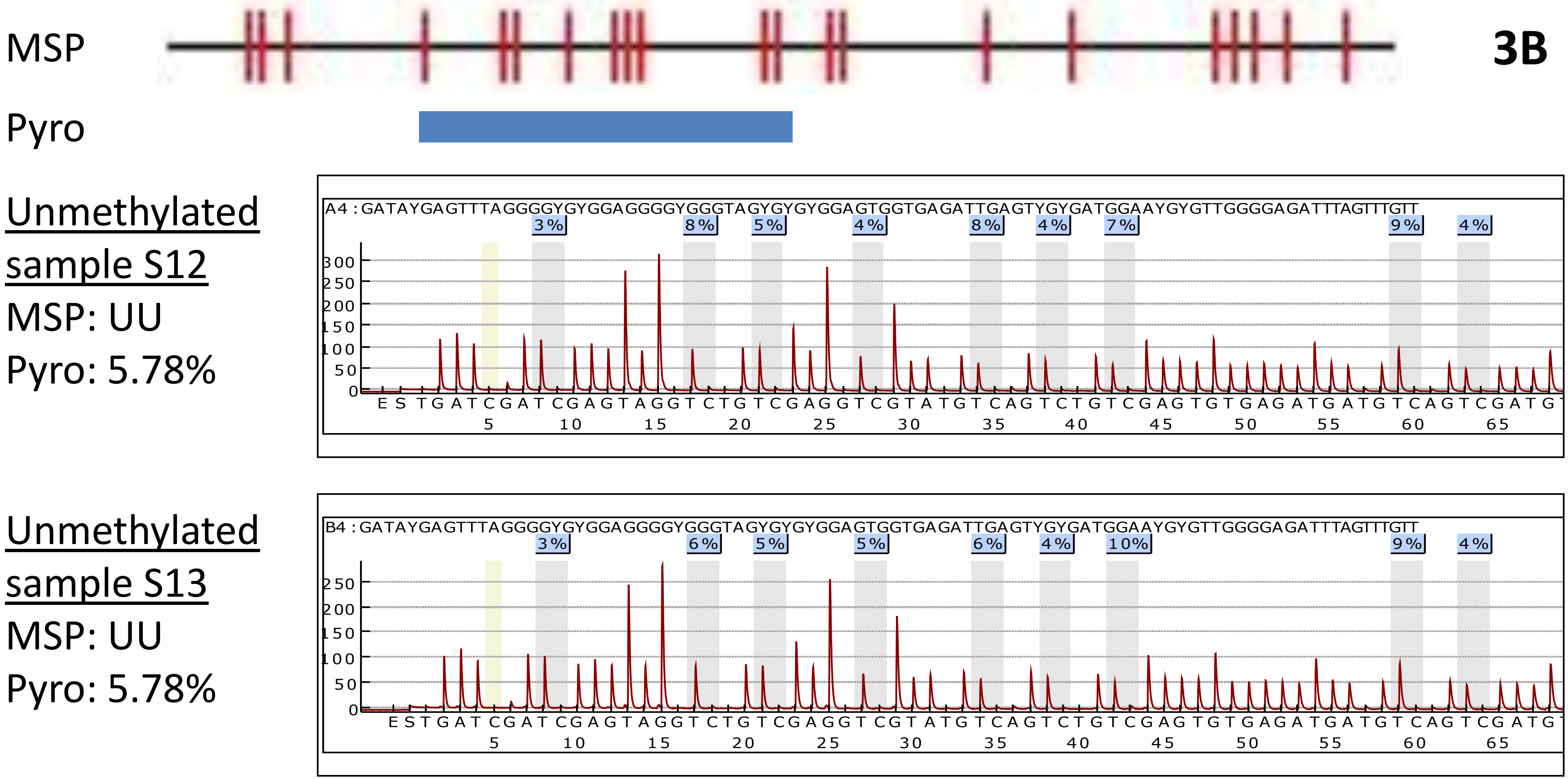

Additional Figure 3. Quantitative bisulfite pyrosequencing of MIR129-2. Pyrograms showing the methylation intensity on a stretch of 9 neighboring CpG dinucleotides of **(A)** methylated primary NHL samples, and **(B)** unmethylated primary NHL samples.

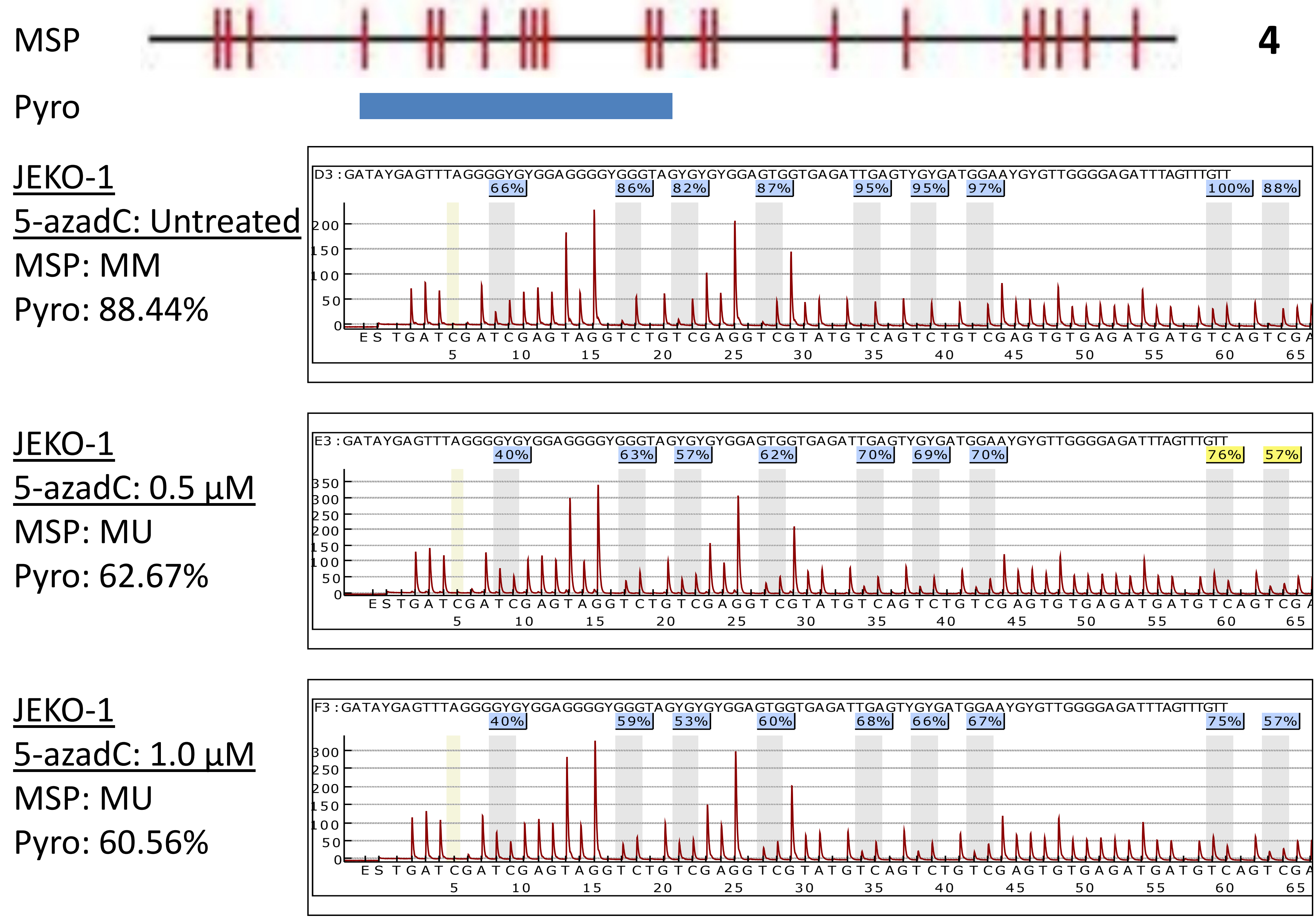

Additional Figure 4. Quantitative bisulfite pyrosequencing of MIR129-2. Pyrograms showing the methylation intensity on a stretch of 9 neighboring CpG dinucleotides of JEKO-1 cells before and after 5-azadC treatment.
